# The Role of Nitric Oxide-Induced *ATILL6* in Growth and Disease Resistance in *Arabidopsis thaliana*

**DOI:** 10.3389/fpls.2021.685156

**Published:** 2021-07-02

**Authors:** Murtaza Khan, Tiba Nazar Ibrahim Al Azawi, Anjali Pande, Bong-Gyu Mun, Da-Sol Lee, Adil Hussain, Byung-Hyun Lee, Byung-Wook Yun

**Affiliations:** ^1^School of Applied Biosciences, Kyungpook National University, Daegu, South Korea; ^2^Ministry of Agriculture State Company for Agricultural Supplies, Baghdad, Iraq; ^3^Department of Entomology, Abdul Wali Khan University Mardan, Mardan, Pakistan; ^4^Division of Applied Life Science (BK21 Program), Institute of Agriculture and Life Science (IALS), Plant Molecular Biology and Biotechnology Research Center (PMBBRC), Gyeongsang National University, Jinju, South Korea

**Keywords:** *Arabidopsis thaliana*, growth under control, oxidative stress, nitro-oxidative stress, plant defense

## Abstract

Nitric oxide (NO) is a signaling molecule that regulates various processes, including plant growth and development, immunity, and environmental interactions. Using high throughput RNA-seq data, we explored the role of the NO-induced *ATILL6* gene in plant growth and defense using functional genomics. The *atill6* mutant and wild-types were challenged with either oxidative (H_2_O_2_, MV) or nitro-oxidative (CySNO, GSNO) stress conditions, and the phenotypic results showed that *ATILL6* gene differentially regulates cotyledon development frequency (CDF) as well as the root and shoot lengths of the plants. To investigate whether *ATILL6* plays a role in plant basal or *resistance* (*R*)*-gene*-mediated defense, the plants were challenged with either virulent or avirulent strains of *Pseudomonas syringae* pathovar tomato (Pst) DC3000. The *atill6* line showed a susceptible phenotype, higher pathogen growth, and highly reduced transcript accumulation of *PR1* and *PR2* genes. These results suggested that *ATILL6* positively regulates plant basal defense. Furthermore, after the inoculation of *atill6* with avirulent Pst (DC3000), the expressions of the *PR1* and *PR2* genes decreased, suggesting a positive role in *R-gene*-mediated resistance in protecting the plant from further spread of disease. We also investigated the role of *ATILL6* in systemic acquired resistance (SAR), and the results showed that *ATILL6* positively regulates SAR, as the mutant line *atill6* has significantly (*p* ≤ 0.05) lower transcript accumulation of *PR, G3DPH*, and *AZI* genes. Overall, these results indicate that the NO-induced *ATILL6* gene differentially regulates plant growth and positively regulates plant basal defense, *R-gene*-mediated resistance, and SAR.

## Introduction

Plants, due to their non-motile nature, are continuously subjected to biotic and abiotic stresses. This exposure induces the production of reactive oxygen species (ROS) and reactive nitrogen species (RNS) in plants, which are key signaling molecules (Domingos et al., 2015; Farnese et al., 2016; Khan et al., 2019). Due to the versatile role of Nitric oxide (NO) it was named as the “Molecule of the year” by the Science magazine in 1992 (Culotta and Koshland, 1992). Afterwards, in 1998 the Nobel Prize in Physiology or Medicine was awarded to Robert F. Furchgott, Louis J. Ignarro, and Ferid Murad jointly for their work on nitric oxide as a signaling molecule in the cardiovascular system. Although NO has been thoroughly studied in animals, its production was reported in plants in 1979 (Klepper, 1979). The organelles involved in NO production in plants are chloroplasts, peroxisomes, mitochondria, apoplasts (Roszer, 2012), endoplasmic reticula, and cell membranes (Fröhlich and Durner, 2011). NO is produced in plants by both enzymatic and non-enzymatic processes, which mainly include the oxidative and reductive release of NO (Khan et al., 2014). NO synthase has been identified in animals and other organisms, but it has not yet been identified in plants (Roszer, 2012). In plants, NO is produced by nitrate (NO_3_-) reductase (NR) activity on NO_3_- and nitrite (Rockel et al., 2002). The production of NO by NR is involved in different biological processes, such as plant immunity and environmental interactions (Mur et al., 2013). Hormones, chemicals, environmental interactions, and osmotic stress play stimulating roles in NO production in plants (Bright et al., 2006; Talwar et al., 2012; Wang et al., 2014). NO is also involved in plant growth and development (Sanz et al., 2014). Recently, the role of NO in plants has been extensively studied in both biotic (Hong et al., 2008; Yun et al., 2012; Khan et al., 2019; Rolly et al., 2020) and abiotic stress conditions (Cantrel et al., 2011; Camejo et al., 2013). To cope with pathogens/pests plants possess various defense mechanisms such as, pathogen associated molecular patterns (PAMPS)-triggered immunity (PTI, formerly called basal resistance), effector-triggered immunity [ETI, formerly called resistance (*R*)-*gene*-mediated], and SAR (Zipfel and Felix, 2005; Jones and Dangl, 2006). Furthermore, in response to pathogens, plants also rapidly produce ROS and RNS, which act as signaling molecules to activate several regulatory pathways (Burniston and Wilson, 2008). To explore the role of NO in plants, new technologies and techniques have been developed. NO can regulate protein function through a post-translational modification called nitrosation; a process through which NO reacts covalently with the cysteine (CyS-Fe-NO) residues of the target proteins (Wendehenne et al., 2001; Hess et al., 2005). The reservoirs of NO in the cells are glutathione and CyS-Fe-NO, which are degraded through cellular functions as and where required (Wendehenne et al., 2001; Graziano and Lamattina, 2005). In studies related to plants, the most commonly used NO donors are S-nitrosocysteine (CySNO), S-nitrosoglutathione (GSNO/SNOG) (Askew et al., 1995), and sodium nitroprusside (SNP) (Bivalacqua et al., 1999). Of these, CySNO and GSNO are preferred because they spontaneously release NO (Uehara et al., 2006; Cho et al., 2009). After infiltration into the plants, NO causes S-nitrosation, a post-translational modification of proteins (Zhu et al., 2008). For NO homeostasis, there are several scavengers, including CPTIO (Hogg et al., 1995), 2-4-carboxyphenyl-4,4,5,5-tetramethylimidazoline-1-oxyl-3-oxide (Amano and Noda, 1995), DTCS (Doi et al., 1996), and MGD (Komarov and Lai, 1995).

Plants possess an efficient antioxidant system for the homeostasis of ROS (Rao and Puppo, 2009; Mittler et al., 2011). In *Arabidopsis* ILR1-like (ILL) is a seven-gene family consisting of *ILL1* (At5g56650), *ILL2* (At5g56660), *ILL3* (At5g54140), *IAR3* (*ILL4*) (At1g51760), *ILL5* (At1g51780), *ILL6* (At1g44350), and *ILR1* (*ILL7*) (At3g02875). In this family, the first gene to be described was *IAR3* (*ILL4*), which encodes an indole acetic acid-alanine (IAA-Ala) hydrolase (Davies et al., 1999), a wound, and a jasmonate-induced gene named *Jasmonate Responsive 3*, and is used as a robust JA pathway marker (Titarenko et al., 1997). Auxins play a vital role in the growth and development of plants, but homeostasis of this hormone remains unknown. One important process of auxin homeostasis is the conjugation of the auxin IAA. The auxin conjugates that have been investigated in *Arabidopsis thaliana* seedlings are IAA-Leu, IAA-Ala, IAA-Asp, IAA-Glu, and IAA-Glc (Kowalczyk and Sandberg, 2001). These conjugates have several key functions in plants, including storage, transport, and the inactivation of IAA (Bartel et al., 2001). The auxin conjugates are converted into indole-3-acetate by the action of several enzymes, including IAR3, ILL5, and ILL6, and are eventually converted by several chemical reactions into camalexin (Truman et al., 2010). Camalexin is a secondary metabolite and is a vital phytoalexin that plays a crucial role during biotic stress in *A. thaliana* (Glawischnig, 2007). It is important to mention that in an earlier investigation involving transcriptomic analysis of *Arabidopsis thaliana* (Hussain et al., 2016) we found that the transcript accumulation of ILL6 increased by 97% following 1mM CySNO treatment. Therefore, in the current study, we investigated the role of the NO-induced *ATILL6* (IAA-leucine resistant (ILR)-like gene) in plant growth and development under control, oxidative (H_2_O_2_ and MV), and nitro-oxidative (CySNO and GSNO) stress conditions. In addition, we investigated the role of NO-induced *ATILL6* in plant basal defense, *R-gene*-mediated resistance, and systemic acquired resistance (SAR). For this purpose, the loss-of-function mutant *atill6* and relative control genotypes were inoculated with the virulent and avirulent pathogenic bacteria *Pseudomonas syringae* (DC3000).

## Materials and Methods

### Plant Materials and Growth Conditions

Seeds of the *A. thaliana* wild-type (WT) ecotype columbia zero (Col-0) and the loss-of-function mutant lines *atill6* (At1G44350), *atgsnor1-3, atcat2*, and *atsid2* were obtained from Nottingham Arabidopsis Stock Center (http://arabidopsis.info/). The *atill6* T-DNA insertion mutant line stock number SALK_22342.42.60.x in Col-0 background was ordered. The seeds were sown under long-day conditions (16 h light and 8 h dark) on either 1/2 Murashige and Skoog (MS) medium or soil at 23 ± 2°C. At the rosette stage (4-week-old plants), samples were collected for genotyping through PCR for the confirmation of T-DNA insertion. The *atill6* mutant plants were genotyped to verify T-DNA insertion in the gene of interest (Supplementary Figure 1). The PCR products were sequenced to confirm T-DNA insertion (Supplementary Material). Furthermore, RTPCR was also performed to verify the abolishment of *ATILL6* expression in the mutant line (Supplementary Figure 2). All genotypes used in this study were of the Col-0 background. The *atgsnor1-3* line was used as a sensitive control. GSNOR1 has a well-established role in plant multiple developmental programs and plant immunity (Kwon et al., 2012). It is a representative line for studying Arabidopsis responses under variable nitro-oxidative environments. The *atcat2* was used as a sensitive control for oxidative stress. CAT2 is a leaf, root and seed-expressed Class I catalase with significantly higher transcript abundance than the other catalases and shows circadian and photosynthetic-type rhythm in Arabidopsis (Zhong et al., 1994; Mhamdi et al., 2010). CAT2 is responsible for most of the catalase activity in Arabidopsis as knockout lines of *cat1* and *cat3* show much less decrease in leaf catalase activity than *cat2* (Mhamdi et al., 2010. Journal of Experimental Botany, https://doi.org/10.1093/jxb/erq282). Therefore, ca*t2* line is regularly used as an oxidative stress-mimic model. For the salicylic acid (SA) pathway, *atsid2* knockout mutant was used (Kim et al., 2013). The Salicylic Acid Induction Deficient 2 (SID2) encodes Icochorismate Synthase 1 (ICS1). The *atisid2* mutant fails to accumulate SA and is deficient in SA-depenent defense responses.

### Oxidative and Nitro-Oxidative Stress Conditions

To explore the role of *ATILL6* under redox stress, plants were subjected to oxidative hydrogen peroxide (H_2_O_2_) and methyl viologen (MV) and nitro-oxidative stresses (CySNO and GSNO) based on the methods described previously (Khan et al., 2019). The seeds were surface sterilized in 50% commercial bleach with 0.1% Triton X-100 (Sigma Aldrich, USA) for 5 min. The seeds were then rinsed three times with sterilized distilled water and stratified at 4°C for 24 h. The seeds were sown on ½ MS medium supplemented with either 2 mM of H_2_O_2_ or 1 μM of methyl viologen for oxidative stress and 0.75 mM of GSNO and 0.75 mM of CySNO for nitro-oxidative stress. After 2 weeks, results were obtained for cotyledon development frequency (CDF) and root and shoot lengths with at least three replicates per treatment as previously described (Shahid et al., 2019). The CDF was used for green developed seedlings (Yun et al., 2011).

### Pathogen Growth and Inoculation and Electrolyte Leakage Assay

The virulent and avirulent strains of *P. syringae* pv. tomato (Pst) DC3000 were grown and inoculated, as described previously (Yun et al., 2011). The bacterial strains were grown on Lauria–Bertani (LB) agar media supplemented with appropriate antibiotics for selection and incubated at 28°C overnight. The single colony was transferred to LB broth with appropriate antibiotics and incubated overnight at 28°C with continuous shaking. The strains were harvested by centrifugation at 8,000 rpm for 3 min and resuspended in 10 mM of MgCl_2_. To explore, the role of ATILL6 in the basal defense and *R-*gene-mediated resistance, the *atill6* line along with the WT and other relevant controls; *atgsnor1-3* and *atsid2*, were inoculated with virulent *Pst* DC3000 or avirulent *Pst* DC3000 with the *avr*B effector protein. The strains were infiltrated into the abaxial side of the leaves at a concentration of 5 × 10^5^ colony-forming units (CFU). The control plants were infiltrated with only 10 mM of MgCl_2_. Leaf samples were collected at designated time points for the expressions of *PR* genes. To investigate further, we evaluated the pathogen growth in the WT and *atill6* along with the relevant control mutants. The electrolyte leakage was measured as described previously (Dellagi et al., 1998) with slight modifications. The designated time points for the measurement of conductivity were 1, 2, 4, 6, 8, 12, and 24 h post-infiltration using a portable conductivity meter (HURIBA Twin Cond B-173, Japan).

### Quantitative Real-Time PCR (qRT-PCR) Analysis

The total RNA extraction and qRT-PCR analyses were performed as described previously (Hussain et al., 2016). Briefly, total RNA was extracted from the inoculated leaves using TRIzol reagent (Invitrogen, USA) according to the manufacturer's instructions. Complementary DNA (cDNA) was synthesized using the DiaStar™ RT kit (SolGent, Korea) according to the manufacturer's instructions. For transcript accumulation analysis, cDNA was used as a template in the EcoTM real-time PCR machine (Illumina, USA) using the 2X Real-time PCR Master Mix (including SYBR® Green I BioFACT™, Korea) along with 100 ng of template DNA and 10 nM of each primer to a final volume of 20 mL. As a negative control, No Template Control was used, which contains only distilled water instead of template DNA. A two-step PCR reaction was established for 40 cycles under the following conditions: polymerase activation at 95°C for 15 min, denaturation at 95°C for 15 s, and annealing and extension at 60°C for 30 s. The melting curves were assessed at 60–95°C for the verification of amplicon specificity for each primer pair, and actin was used as an internal reference gene (Shahid et al., 2019). The primers used in this study are listed in Supplementary Table 1.

### Statistical Analysis

For all assays, the experiments were performed more than twice and the representative results are presented. In media stress conditions, the data point is the mean of three replicates with five plants pooled in each replicate, while for the pathogenicity assay, the data point is the mean of three replicates. The significant difference between each treatment were analyzed by one-way ANOVA analysis of varience, followed by Duncan's multiple range test using statistical analysis system (SAS 9.1). The mean values, standard deviations, and standard errors were obtained in the Microsoft Excel program. The data were then visualized using GraphPad Prism software (version 6.0, San Diego, CA, USA).

## Results

### *ATILL6* Differentially Regulates Root and Shoot Length Under Oxidative and Nitro-Oxidative Stress Conditions

**Figure 1:**
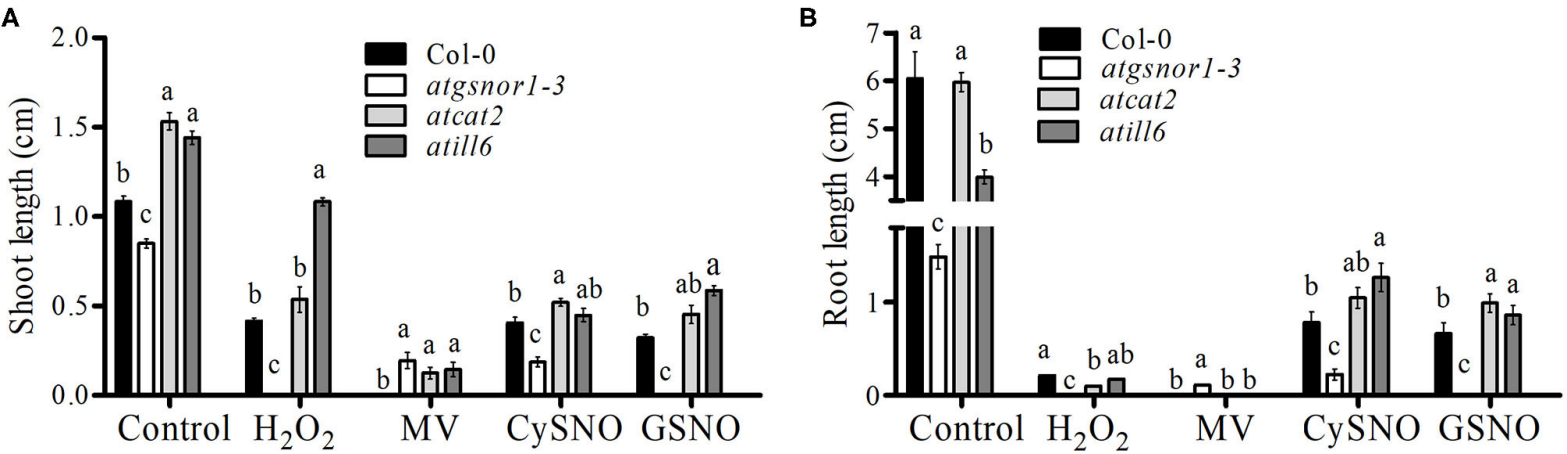
The response of *atill6* and relevant control plants after exposure to oxidative (H2O2 and MV), and nitrosative (CySNO and GSNO) stress conditions. **(A)** Shoot and **(B)** Root lengths. All data points show the mean of at least three replicates, and the experiment was repeated twice with similar results. The significant difference between the treatments is represented by (a, b, c) one-way ANOVA analysis of varience, followed by Duncan's multiple range test using statistical analysis system (SAS 9.1).

To investigate the role of *AtILL6* in plant growth and development, the assessed growth parameters were: CDF, shoot and root length. The *atgsnor1-3* (deficient in S-nitrosoglutathione reductase enzyme -GSNOR1), and *atcat2* (deficient in the CATALASE2 enzyme) were used as control plants due to their established role in plant growth and defense (Feechan et al., 2005a; Hu et al., 2010). There was no difference between the CDFs of *atill6, atgsnor1-3*, and *atcat2* under control conditions compared to that of the WT (Supplementary Figure 3). In contrast, under H_2_O_2_ (oxidative stress) conditions, *atill6* showed a significant increase (*p* ≤ 0.05) in CDF compared to that of the WT, but no significant difference was observed under MV (Supplementary Figure 3). On the other hand, under nitro-oxidative stress conditions (CySNO and GSNO), the CDF in *atill6* significantly increased compared to that of the WT (Supplementary Figure 3). The shoot length of the *atill6* was significantly longer (*p* ≤ 0.01) under control, oxidative (H_2_O_2_ only), and nitro-oxidative stress conditions induced by CySNO and GSNO as compared to that of the WT plants (Figure 1A and Supplementary Figure 4). The root length of *atill6* was significantly shorter under control conditions, but a significant increase was recorded under both CySNO and GSNO stress conditions compared to that of the WT (Figure 1B and Supplementary Figure 4).

### ATILL6 Positively Regulates Plant Basal Defense

**Figure 2:**
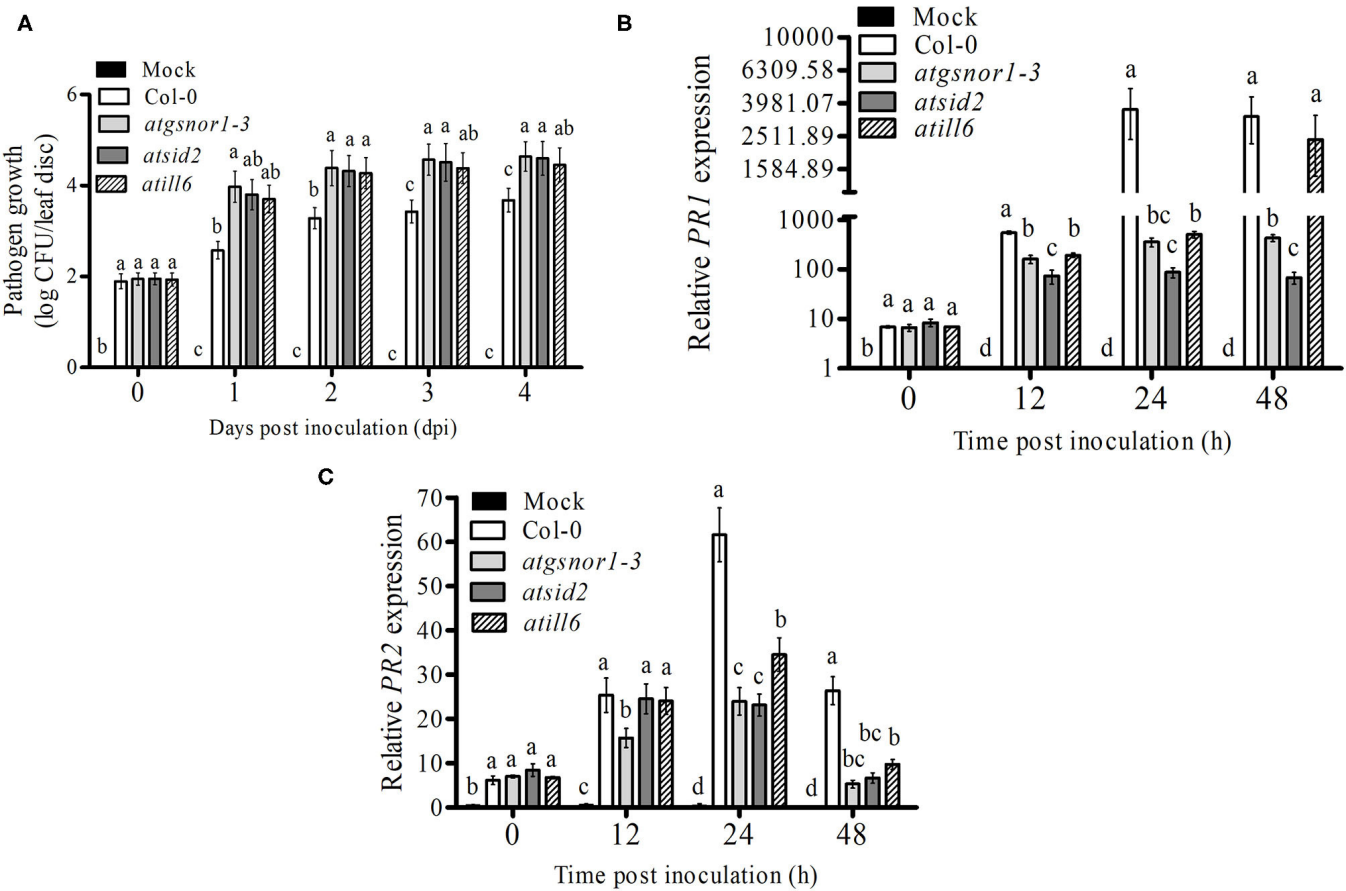
*ATILL6* positively regulates the plant basal defense. **(A)** pathogen growth and **(B,C)** Relative gene expressions of *PR1* and *PR2* genes from infiltrated leaves in the indicated genotypes after inoculation with *Pst* DC3000 virulent bacteria. All data points are the means of three replicates, and error bars represent ± standard error. The significant difference between the treatments is represented by (a, b, c, d), one-way ANOVA analysis of varience, followed by Duncan's multiple range test using statistical analysis system (SAS 9.1).

To explore the role of AtILL6 in the plant basal defense system, the *atill6* line along with WT and genotypes, *atgsnor1-3* and *atsid2* were inoculated with virulent *Pst* DC3000. Previously, it has been reported (Feechan et al., 2005b), that *atgsnor1-3* is susceptible to *Pst* DC3000. In the present study, we found that *atill6* showed a susceptible phenotype compared to that of the WT (Supplementary Figure 5). To investigate further, we evaluated the pathogen growth in the WT and *atill6* along with the relevant control mutants. No significant difference was observed in any genotype for bacterial growth at 0 days post-inoculation (dpi), but at 1, 2, 3, and 4 dpi, *atill6* showed a significant increase (*p* ≤ 0.05) in pathogen growth compared to that of the WT (Figure 2A). These results indicate that ATILL6 plays a positive role in the basal defense system of the plants. As the response shown by plants to biotroph pathogens is induced by a significant plant hormone SA, we investigated the role of ATILL6 in the SA pathway and aimed to quantify the transcript accumulation of *PR1* and *PR2*, the important marker genes in this pathway, in all genotypes used in this study. The results of qRT-PCR revealed significantly lower (*p* ≤ 0.05) expression of *PR1* genes in *atill6* after 12, and 24 h, as compared to that of the WT though no significant difference was observed after 48 h of inoculation (Figure 2B). Furthermore, a significant decrease (*p* ≤ 0.05) was observed in the expression of the *PR2* gene at 24 and 48 h, but not at the 12 h time pint (Figure 2C). The other mutants *atgsnor1*-3 and *atsid2* also showed reduced PR1 and PR2 transcript accumulation compared to that of the WT at all-time points (Figures 2B,C).

### ATILL6 Positively Regulates *R-gene*-Mediated Resistance

**Figure 3:**
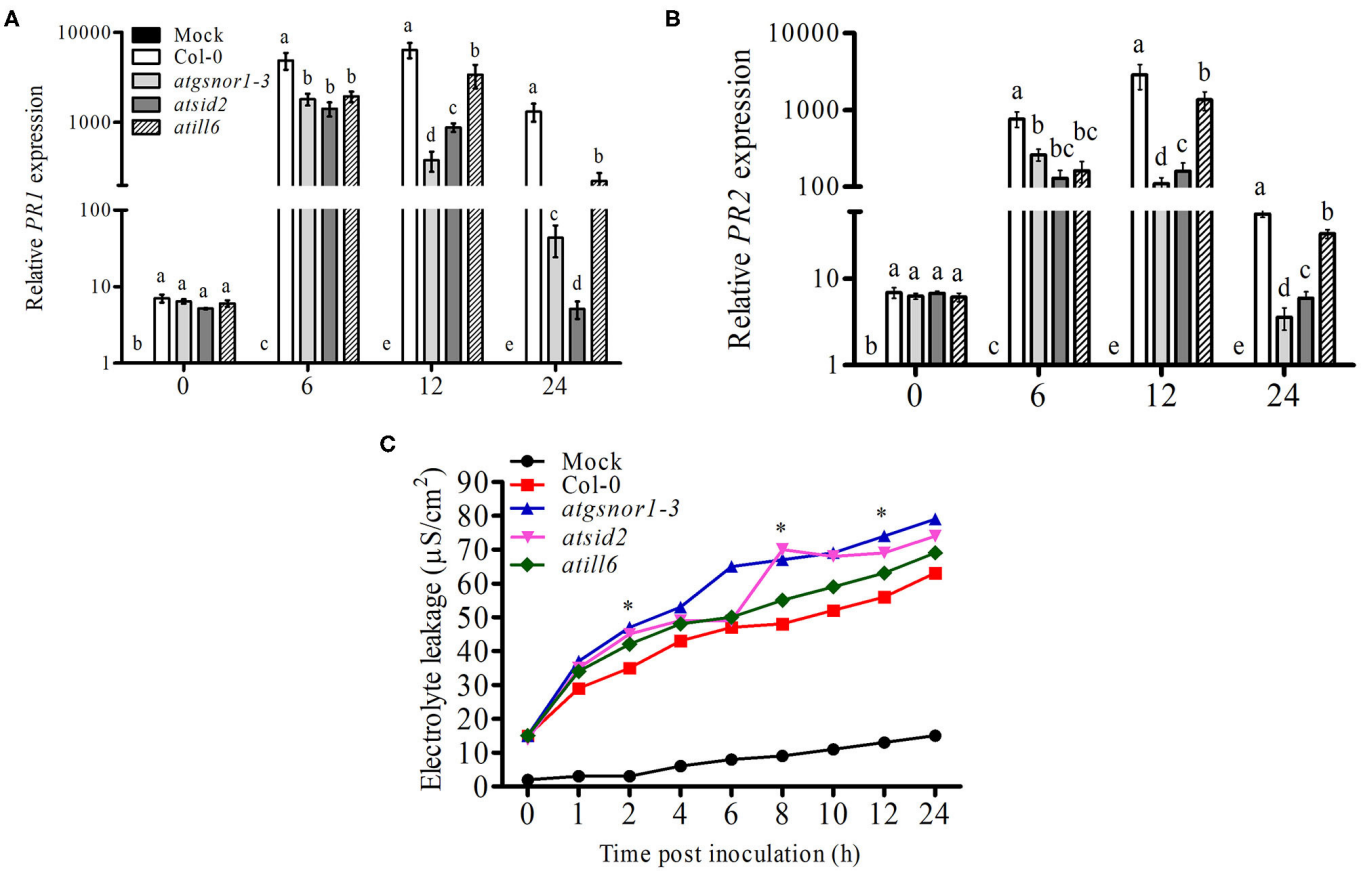
*ATILL6* positively regulates plant R-gene-mediated resistance. **(A,B)** Relative gene expression of *PR1* and *PR2* genes and **(C)** electrolyte leakage in the indicated genotypes after inoculation with *Pst* DC3000 avirulent bacteria. All data points are the means of three replicates, and error bars represent ± standard error. The significant difference between the treatments is represented by (a, b, c, d, e), one-way ANOVA analysis of varience, followed by Duncan's multiple range test using statistical analysis system (SAS 9.1).

Plants recognize pathogen-released effector molecules by using *R-genes* encoded by the nucleotide-binding site leucine-rich repeats to induce *R-gene*-mediated resistance (Jones and Dangl, 2006). Following *R-gene*-mediated resistance, plant cells intentionally commit a type of cell suicide called the hypersensitive response (HR) to prevent further spread of disease (Jones and Dangl, 2006). Therefore, we further investigated the functional role of ATILL6 in *R-gene*-mediated resistance and the HR. For this purpose, all genotypes were challenged with an avirulent strain of *Pst* DC3000 with the *avr*B effector protein. After inoculation, the samples were collected from all genotypes at designated time points (0, 6, 12, and 24 h) to analyze the *PR* gene expression. The qRT-PCR results revealed a significant reduction (*p* ≤ 0.05) in the transcript accumulation of *PR1* in *atill6* after 6, 12, and 24 h. Similarly, a significant reduction was recorded in the expression of *PR2* genes after 6 and 12 h post-inoculation compared to that of the WT (Figures 3A,B). Additionally, *atill6* had higher levels of electrolyte leakage over time compared to that of the WT (Figure 3C). These results indicate that ATILL6 plays a positive role in *R-gene*-mediated resistance, a defense system of plants. Reduced *PR1* and *PR2* transcript accumulation was also observed for both *atsid2* and *atgsnor1*-3 (Figures 3A,B).

### ATILL6 Positively Regulates SAR

**Figure 4:**
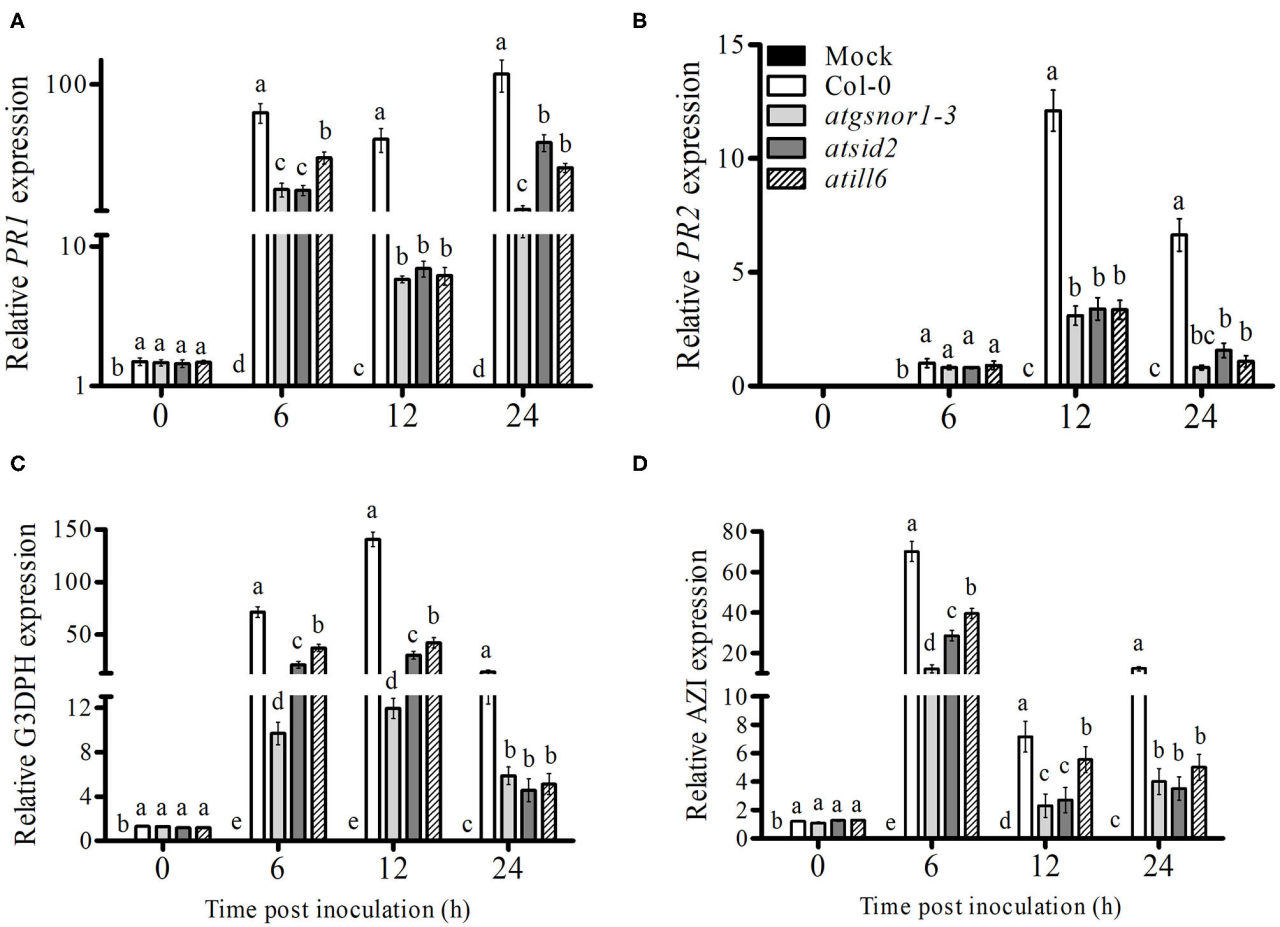
*ATILL6* positively regulates plant systemic acquired resistance. **(A–D)** Relative gene expression of *PR1, PR2, G3DPH*, and *AZI* genes, respectively, in the systemic leaves of the indicated genotypes after inoculation with *Pst* DC3000 avirulent bacteria. All data points are the means of three replicates, and error bars represent ± standard error. The significant difference between the treatments is represented by (a, b, c, d, e), one-way ANOVA analysis of varience, followed by Duncan's multiple range test using statistical analysis system (SAS 9.1).

SAR, a vital defense system of plants, is induced after local infection in the uninfected areas of plants. Both ROS and RNS are involved in the induction of SAR (Song et al., 2006). Therefore, we investigated the role of ATILL6 in SAR. For this purpose, plants were challenged with *Pst* DC3000 (avrB) at 5 × 10^6^ CFU. After pathogen inoculation, samples were collected from non-inoculated leaves (systemic leaves) and the transcript accumulation of important SAR marker genes, such as *PR1, PR2*, glyceraldehyde 3-phosphate dehydrogenase, and azelaic acid inducer (*AZI*) were analyzed over time. The *PR1* gene transcript accumulation was significantly (*p* ≤ 0.05) and highly significantly (*p* ≤ 0.01) lower after 6 and 12 h, and 24 h, respectively, in the systemic leaves of *atill6*, while the *PR2* gene expression was significantly lower after 12 and 24 h compared to that of the WT (Figures 4A,B). Furthermore, *atill6* showed a highly significant and significant decrease in the transcript accumulation of *G3DPH* after 6 and 12 h, and 24 h, respectively (Figure 4C). The expression of AZI significantly decreased after 6, 12, and 24 h, as shown in Figure 4D. The qRT-PCR results implied that ATILL6 plays a positive role in the activation of SAR when challenged with the avirulent pathogen *Pst* DC3000 (*avrB*) at 5 × 10^6^ CFU.

## Discussion

The small redox-active molecule NO with high diffusivity plays a key role in multiple cellular processes, such as seed germination and the plant response to biotic and abiotic stress conditions (Garcia-Mata and Lamattina, 2002; Yun et al., 2011). NO can regulate the transcriptional machinery of certain genes to control different physiological processes. Global changes in gene expression in response to NO have been studied using microarrays (Parani et al., 2004), RNA-seq (Begara-Morales et al., 2014; Hussain et al., 2016), and qRT-PCR (Huang et al., 2002). The present study explored the functional role of NO-induced *ATILL6* gene in plant growth and development under control, oxidative, and nitro-oxidative stress conditions. *atgsnor1-3* deficient in *AtGSNOR1*, as well as *atcat2* deficient in *AtCATALAE2* were used as control plants due to their established role in plant growth and defense (Feechan et al., 2005a; Hu et al., 2010). The phenotypic results revealed that the loss-of-function mutant *atill6* had significantly longer shoot length and shorter root length as comapred to control, which indicate that *ATILL6* negatively regulates plant shoot length and positively regulates root length (Figures 1A,B and Supplementary Figure 3). Imposition of stress using H_2_O_2_ or CySNO and GSNO, the shoot length of *atill6* significantly longer compared to WT (Figure 1A). The root length of the *atill6* mutant line was also found significantly longer under CySNO and GSNO, as shown in Figure 1B. This phenotypic result implied that *ATILL6* positively regulates root length under control conditions but negatively regulates it under CySNO and GSNO stress conditions, compared to that of the WT (Figure 1B). This may be due to the over production of ROS and RNS which leads to higher sensitivity of plants to oxidative stress (Garcia-Mata and Lamattina, 2002). Kopyra et al. (2004) also suggested that an increase in the shoot length under nitro-oxidative stress (GSNO) may be due to the possible role of NO in seed germination and seedling growth. Furthermore, Beligni and Lamattina (2000) suggested that NO may break seed dormancy to provide a good starting point for plants to grow which may be even better than GA3. To sum up, the current study revealed that *ATILL6* differentially regulates the growth traits in *A. thaliana* under control and oxidative and nitro-oxidative stress conditions, depending upon the type of ROS and RNS donors.

We further evaluated the role of NO-induced *ATILL6* in plant basal defense, *R-gene*-mediated resistance, and SAR. As indicated by Supplementary Figure 4, the loss-of-function mutant line *atill6* plants were susceptible to infection when they were exposed to a virulent pathogen (Pst DC3000). This was further confirmed by the qRT-PCR results of the SA-dependent *PR* gene expressions, and the relative expressions of the *PR1* and *PR2* genes were significantly reduced in the loss-of-function mutant *atill6* compared to those of the WT (Figure 2). The disease-susceptible phenotype, higher pathogen growth, and lower expression of *PR* genes indicated that NO-induced *ATILL6* has a major role in plant basal defense. Therefore, we further evaluated the role of *ATILL6* in ETI. Plants can identify the effector proteins of pathogens, such as *avr*, by the *R-gene* and cause *R-gene*-mediated resistance to reduce further spread of the disease (Glazebrook et al., 1996). The results indicate that, similar to plant basal defense, *ATILL6* also positively regulated plant *R-gene*-mediated resistance. As shown by Figure 3, after the inoculation of an avirulent pathogen (Pst DC3000 expressing *avr*B effector), the expressions of the *PR* genes were highly reduced in the *atill6* loss-of-function mutant compared to those of the WT. After the recognition of effector proteins by the *R-gene* of the host plant, the defense signals pass from local to systemic tissues of the plants due to the expression of *PR, G3DPH*, and *AZI* genes to activate plant SAR (Wang et al., 2014). SAR is a defense strategy of plants that assists with protecting the plants from secondary infections of virulent pathogens (El-Shetehy et al., 2015). Plants require SA for pathogen identification, the subsequent establishment of local resistance, and, eventually to the whole plant in order to protect them against biotic stress conditions (Tsuda et al., 2008). Thus, we also investigated the role of ATILL6 in SAR, and the results revealed that *ATILL6* positively regulates SAR, as the mutant line *atill6* had significantly lower transcript accumulation of *PR, G3DPH*, and *AZI* genes compared to that of the WT (Figures 4A–D).

## Data Availability Statement

The original contributions presented in the study are included in the article/Supplementary Material, further inquiries can be directed to the corresponding author/s.

## Author Contributions

B-WY and MK designed the experiments. MK performed the experiments and wrote the manuscript. AP and TNIAA drafted the manuscript. B-GM and D-SL conducted data analysis. B-HL and AH critically reviewed and edited the manuscript. B-WY provided supervision. All authors have read and agreed to the published version of the manuscript.

## Conflict of Interest

The authors declare that the research was conducted in the absence of any commercial or financial relationships that could be construed as a potential conflict of interest.

## Supplementary Material

Supplementary Figure 1 Genotyping of *atill6* mutant line.

Supplementary Figure 2 RTPCR confirmation for the abolishment of ILL6 expression in the *ill6* T-DNA insertion mutant line.

Supplementary Figure 3 Cotyledon development frequency (CDF), after treatment with oxidative (H_2_O_2_ and MV), and nitrosative (CySNO and GSNO) stress conditions in *atill6* mutant line and relevant control plants. All data points show the mean of at least three replicates, and the experiment was repeated twice with similar results. The significant difference between the treatments is represented by (a, b, c, d) one-way ANOVA analysis of varience, followed by Duncan's multiple range test using statistical analysis system (SAS 9.1).

Supplementary Figure 4 Phenotypic responses, after treatment with oxidative (H_2_O_2_ and MV), and nitrosative (CySNO and GSNO) stress conditions in *atill6* mutant line and relevant control plants.

Supplementary Figure 5 Symptom development in atill6 and relevant control after inoculation with Pst DC3000 virulent bacteria.
