# Supplementary figures and images for: The Role of Nitric Oxide-Induced ATILL6 in Growth and Disease Resistance in Arabidopsis thaliana
Source: Front Plant Sci. 2021 Jul 2;12:685156. doi: 10.3389/fpls.2021.685156 (PMC8285060; doi:10.3389/fpls.2021.685156)

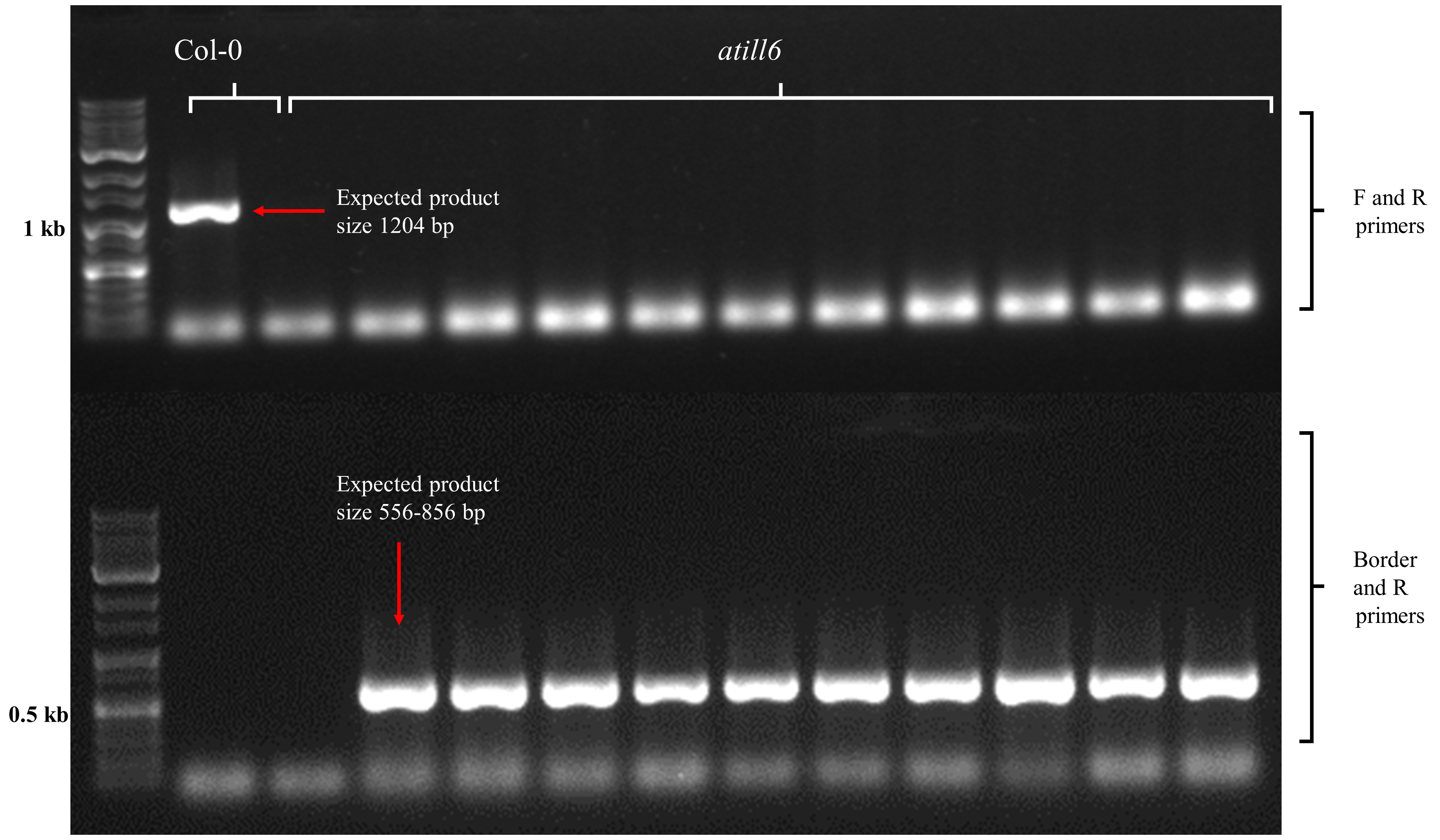

Supplement: Supplementary Figure 1 — Genotyping of atill6 mutant line. [file Image_1.TIF]

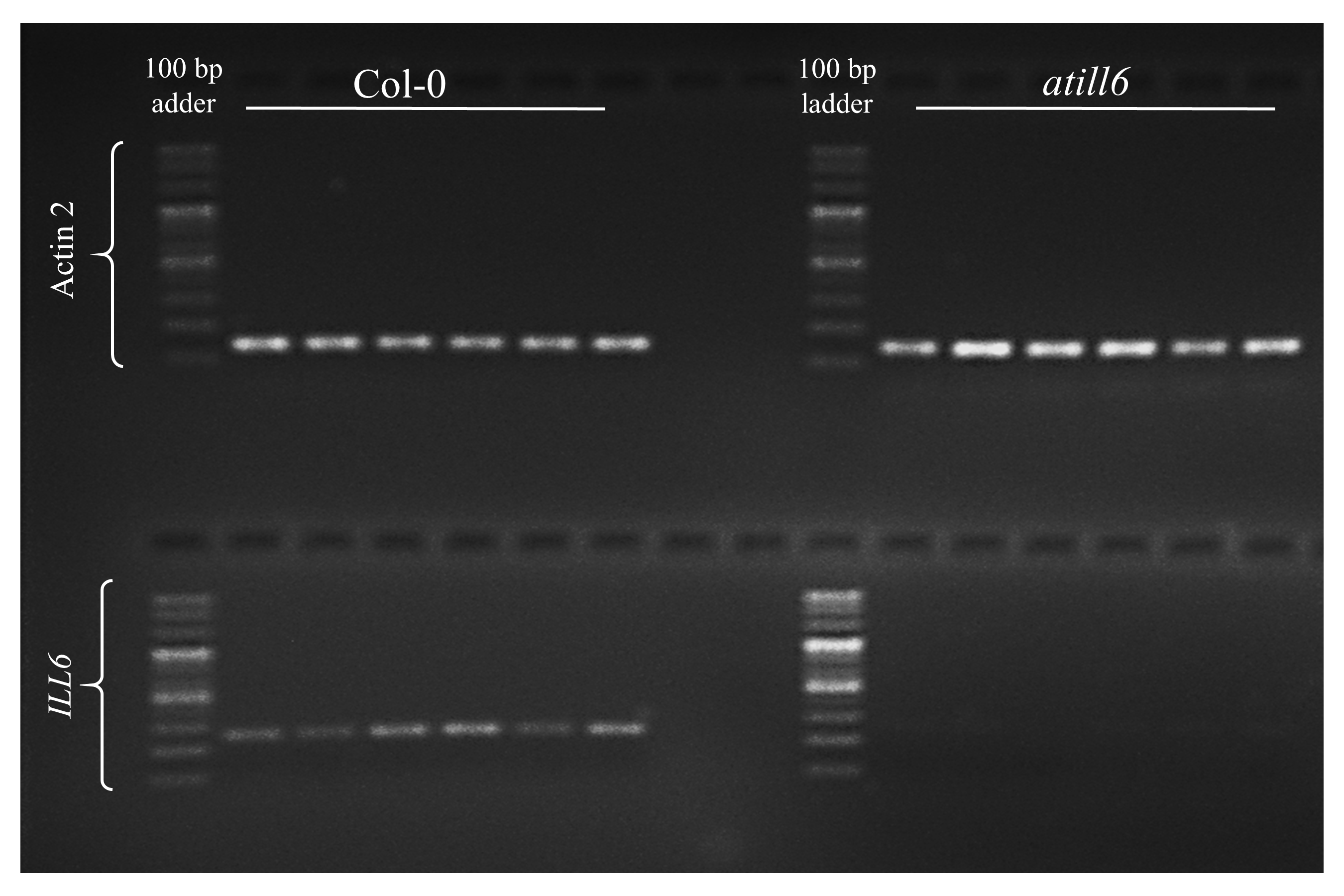

Supplement: Supplementary Figure 2 — RTPCR confirmation for the abolishment of ILL6 expression in the ill6 T-DNA insertion mutant line. [file Image_2.TIF]

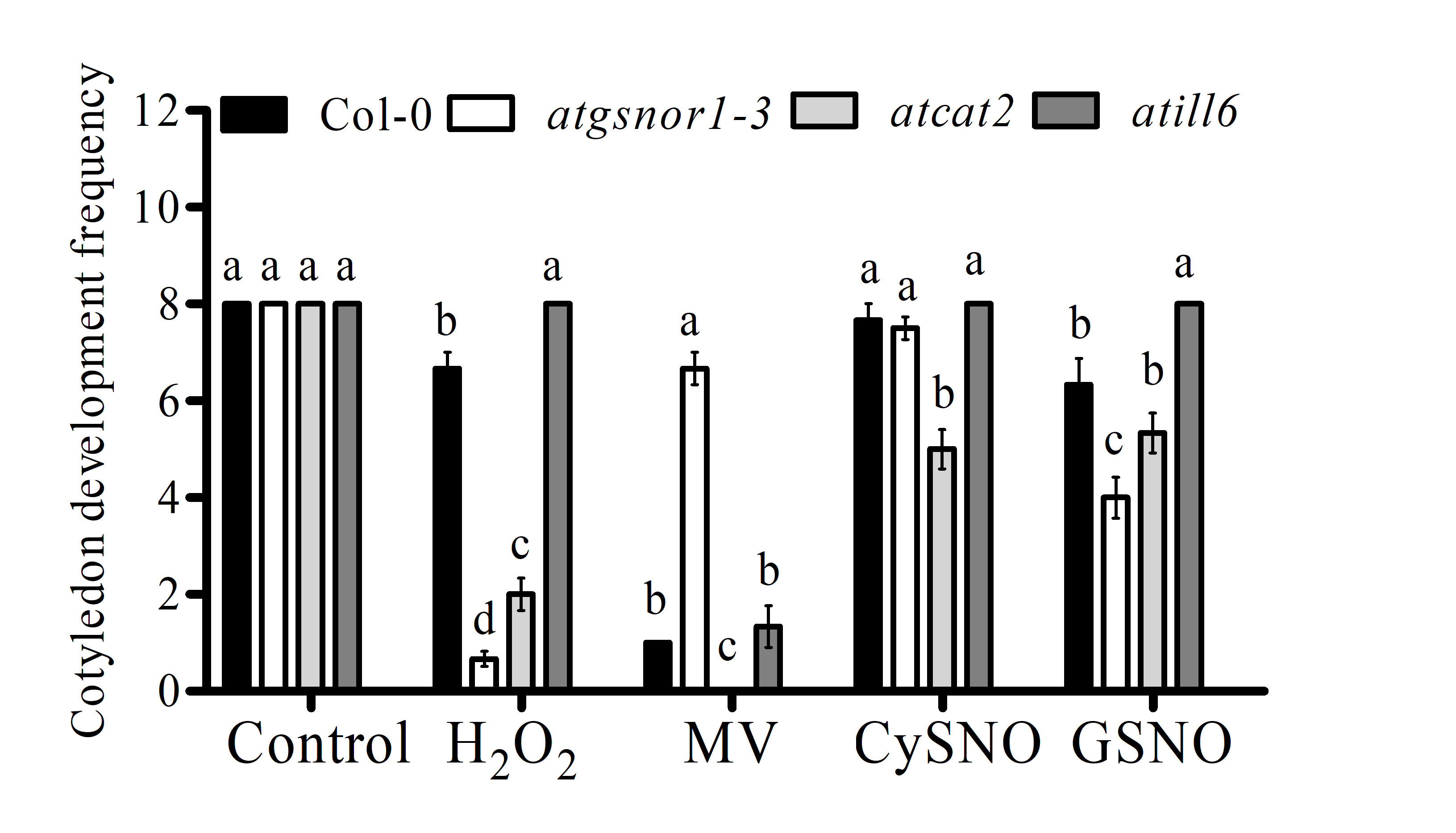

Supplement: Supplementary Figure 3 — Cotyledon development frequency (CDF), after treatment with oxidative (H2O2 and MV), and nitrosative (CySNO and GSNO) stress conditions in atill6 mutant line and relevant control plants. All data points show the mean of at least three replicates, and the experiment was repeated twice with similar results. The significant difference between the treatments is represented by (a, b, c, d) one-way ANOVA analysis of varience, followed by Duncan's multiple range test using statistical analysis system (SAS 9.1). [file Image_3.JPEG]

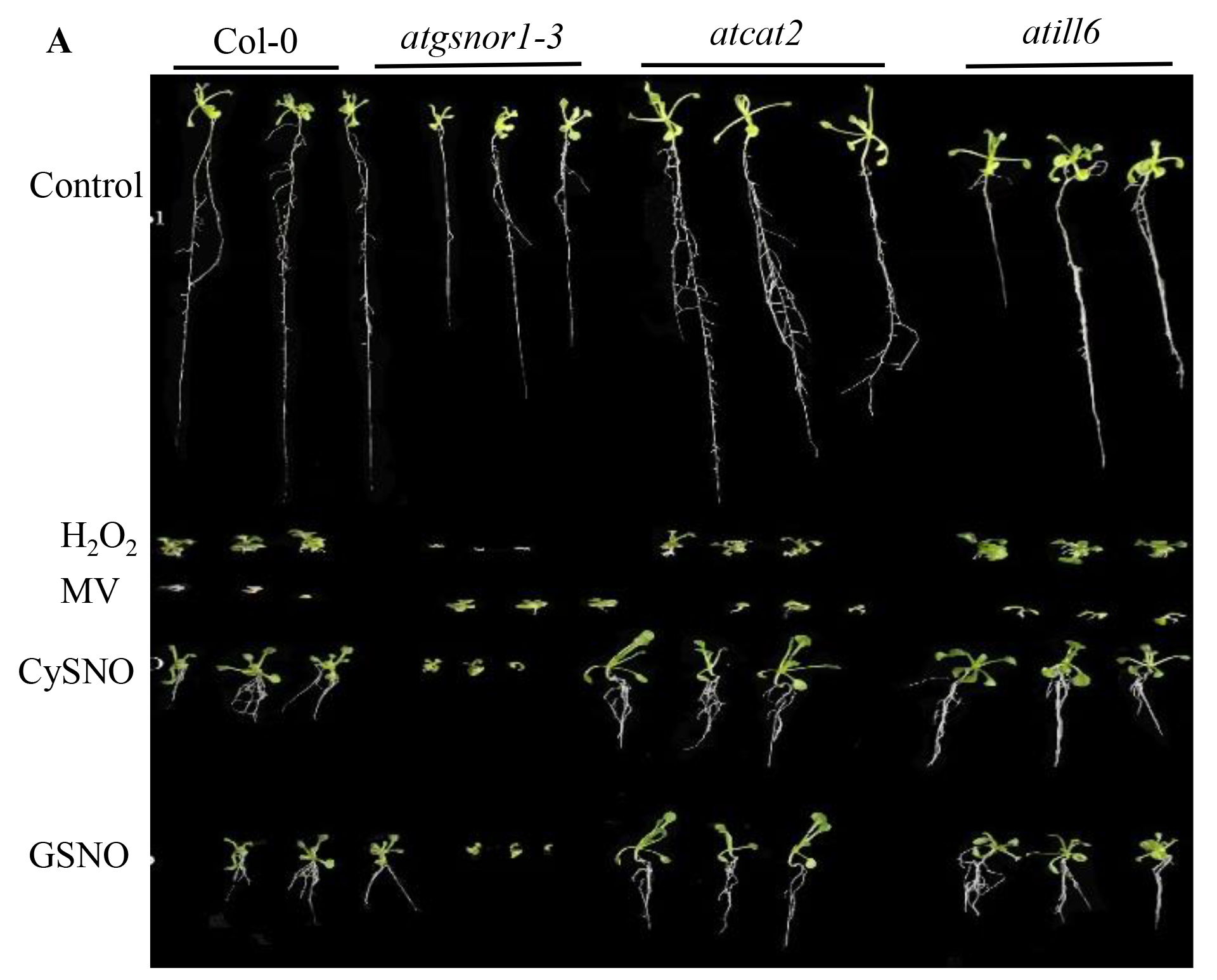

Supplement: Supplementary Figure 4 — Phenotypic responses, after treatment with oxidative (H2O2 and MV), and nitrosative (CySNO and GSNO) stress conditions in atill6 mutant line and relevant control plants. [file Image_4.TIF]

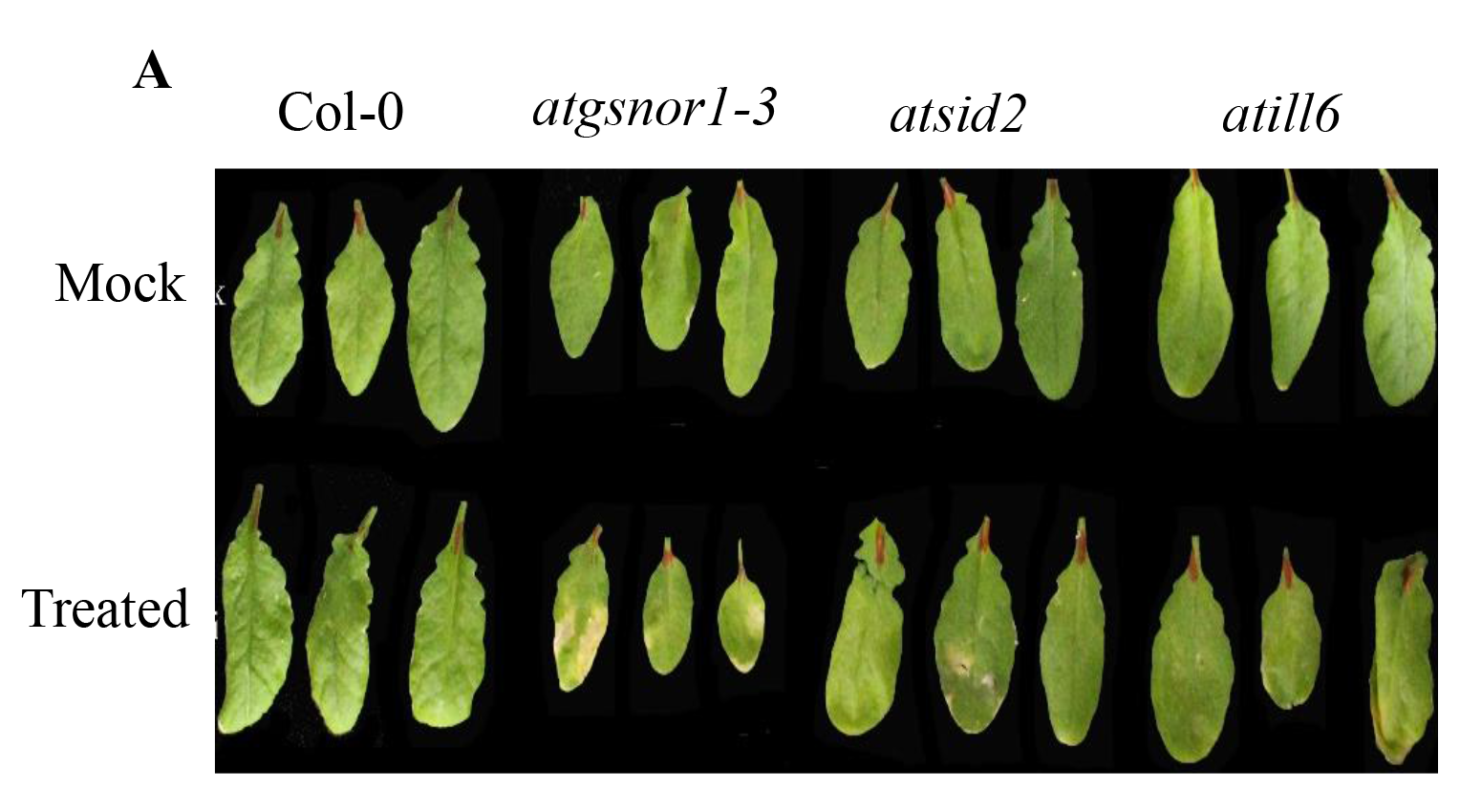

Supplement: Supplementary Figure 5 — Symptom development in atill6 and relevant control after inoculation with Pst DC3000 virulent bacteria. [file Image_5.TIF]
