# Supplementary material for: The Role of Nitric Oxide-Induced ATILL6 in Growth and Disease Resistance in Arabidopsis thaliana
Source: Front Plant Sci. 2021 Jul 2;12:685156. doi: 10.3389/fpls.2021.685156 (PMC8285060; doi:10.3389/fpls.2021.685156)
Supplement: Supplementary file 6 [file Table_1.DOCX]

**Supplementary table 1.** List of primers used in this study.

| S.NO. | Name | Forward primer (5’-3’) | Reverse primer (5’-3’) |
| --- | --- | --- | --- |
|  | *AtILL6* (For Genotyping) | TCCATGTGACCATGTGAAATG | TAGCTTCACGGGATACAATGC |
|  | *PR1* | GTGCAATGGAGTTTGTGGTC | TCACATAATTCCCACGAGGA |
|  | *PR2* | CAGATTCCGGTACATCAACG | AGTGGTGGTGTCAGTGGCTA |
|  | *AZI* | GCAAGCCAAGTCCTAAACCA | GTCGACGTCAACCAAACCTT |
|  | *G3DPH* | CGTCTTTTGGGGAAATCAGA | GACATTGTCAATCGGCACAC |
|  | *AtILL6* (For Expression) | GTGGCGCCGCTAATCTCCT | TTGGCAACCGAAAACCCCGA |
|  | *Actin* | GCTGGACGTGACCTTACTGA | CCATCTCCTGCTCGTAGTCA |
