# Supplementary material for: The Role of Nitric Oxide-Induced ATILL6 in Growth and Disease Resistance in Arabidopsis thaliana
Source: Front Plant Sci. 2021 Jul 2;12:685156. doi: 10.3389/fpls.2021.685156 (PMC8285060; doi:10.3389/fpls.2021.685156)

**Record from TAIR website**

[**https://www.arabidopsis.org/servlets/TairObject?type=polyallele&id=500115805**](https://www.arabidopsis.org/servlets/TairObject?type=polyallele&id=500115805)**)**

[**AT1G44350.1**](https://www.arabidopsis.org/servlets/TairObject?type=gene&id=327890)**-ILL6- SALK-022342C (T-DNA Insertion line)**

**Insertion Flanking sequence**

GTGAATTAATGATTTGCTTTTGAAGATAAGGGAAATCATTTAATTTTTATTTTATTTTTT
TGGTGTAATAGAGGAGTTATGGTCTTTAGGCCCATGGTTTCGTTGGACCGGAGATCAAAG
CATTTATTTTTGTCCCTTTTGTTGCATGTATGATTTTTTTATAGGGAACAGTGGTTCTGT
TATTCCGACCGGCTGAAGAAGCTGGAAATGGTGCCCCCAATGTGATTGTGTGACGGTGCT
TGGGATGACGTGGNGGCTGTCTTCGCGGTCC (Length:271 bp)

**Sequencing Result**

AT1G44350 - ILL6 sequencing performed with SALK left border (LB) primer for confirmation of T-DNA insertion.

CCTATGAAGGCGGTGAGGGCATCAGCTGTTGCCCGTCTCACTGGTGAAAAGAAAAACCACCCCAGTACATTAAAAACGTCCGCAATGTGAATTAATGATTTGCTTTTGAAGATAAGGGAAATCATTTAATTTTTATTTTATTTTTTTGGTGTAATAGAGGAGTTATGGTCTTTAGGCCCATGGTTTCGTTGGACCGGAGATCAAAGCAAATAAATTAGTCCCTTTTGTTGCATGTATGATTTTTTTATAGGGAACAGTGGTTCTGTTATTCCAACCGGCTGAAGAAGCTGGAAATGGTGCAAAGAATATGATCGAAGACGGAGCTTTGGATGACGTGGAGGCTATCTTCGCGGTCCATGTGTCCCATATCCATCCAACGGGTGTGATTGGATCAAGAAGTGGTCCTTTGCTCGCGGGATGTGGAATTTTCCGGGCGGTTATCACTTCGGAAGATAGCCGTGGCGCCGCTAATCTCCTTCTTGCAGCTTCTTCCGCGGTCATTAGTCTTCAAGGCATTGTATCCCGTGAAGCTAA


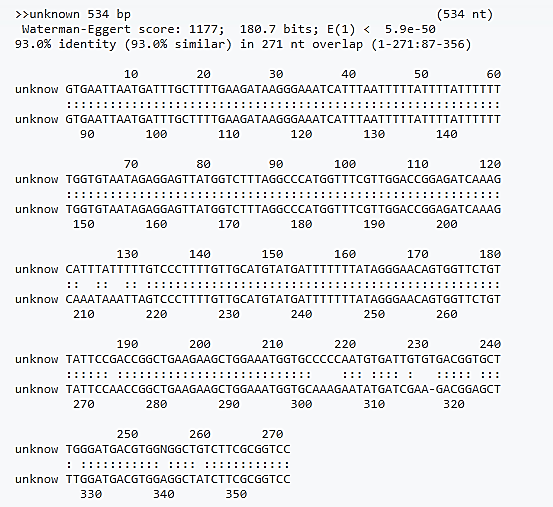

Supplement: Supplementary file 7 [file Data_Sheet_1.docx]
